# Rare Mesenchymal Breast Entities that Mimic Malignancy: A Single-institution Experience of a Challenging Diagnosis

**DOI:** 10.7759/cureus.4000

**Published:** 2019-02-03

**Authors:** Camila C Simoes, Suimin Qiu, Quan D Nguyen, Sandra S Hatch, Jing He

**Affiliations:** 1 Pathology, The University of Texas Medical Branch, Galveston, USA; 2 Pathology, University of Texas Medical Branch, Galveston, USA; 3 Radiology, University of Texas Medical Branch, Galveston, USA; 4 Radiation Oncology, The University of Texas MD Anderson Cancer Center, Galveston, USA

**Keywords:** breast, granular cell tumor, myofibroblastoma, benign mesenchymal tumor of breast

## Abstract

Background

Benign mesenchymal tumors of the breast are rare and may mimic invasive carcinoma on imaging and morphology, thus becoming clinically challenging for clinicians, radiologists, and pathologists. To improve the understanding of these lesions and to avoid erroneous diagnosis and inappropriate treatment, we report our institution’s experience with seven cases of granular cell tumor (GCT) and myofibroblastoma (MFB) in the past 10 years.

Materials and methods

Seven cases of benign mesenchymal tumors of the breast were identified at the University of Texas Medical Branch from 2008 to 2018. Breast biopsies were collected from all patients after mammography and ultrasound imaging classified their results as suspicious or highly suggestive of malignancy by the Breast Imaging Reporting and Data System (BI-RADS ≥ 4A). All cases were reviewed to study the morphologic features and their immunoprofiles. The demographic characteristics, methods of treatment, postoperative pathological results, and follow-up results of the cases were then analyzed and compared to peer-reviewed literature.

Results

The study consisted of five females and two males with a mean age of 50 years in the GCT patients and 62 years in MFB patients. We identified four cases of GCT and three cases of MFB. The mean tumor size was 1.9 cm. Clinically, five patients presented with a palpable nontender mass, one with breast asymmetry, and one was asymptomatic. All patients underwent imaging studies prior to core needle biopsy. BI-RADS was ≥4B in patients with GCT and 4A-C in MFB. Definitive diagnosis was made by histopathology and confirmed by immunohistochemistry in accordance with the features described in the literature. Six patients underwent wide excision. The mean follow-up duration was 44.5 months. All patients remained well, without recurrence.

Conclusions

MFB and GCT can mimic malignant neoplasms and the clinical significance of these entities lies primarily in their recognition as distinctive benign neoplasms. The gold standard for the diagnosis of GCT and MFB is histopathology. All the cases in our series were clinically or radiologically mistaken for carcinoma, which has been largely reported in the literature. Pathologists should bear this in mind to avoid misdiagnosis and unnecessary treatment.

## Introduction

Benign mesenchymal breast entities may mimic invasive carcinoma on imaging and morphology, and their diagnoses can, therefore, be challenging for clinicians, radiologists, and pathologists. Among these entities, mammary myofibroblastoma (MFB) and granular cell tumor (GCT) are two rare lesions that may present as a painless palpable mass or may be clinically asymptomatic and found on routine imaging. On imaging, it can appear as a solid mass or lesion, which is suggestive of malignancy. Despite the advances in multiple imaging modalities, given the variability both within and across them, the accurate diagnosis of these entities remains a tissue diagnosis.

Myofibroblastoma is a rare, benign mesenchymal tumor of the breast. It was first reported in the breast by Wargotz et al. in 1987 [1]. It is composed of spindle cells with myofibroblastic differentiation [2]. Due to the broad morphologic spectrum of MFB, this uncommon benign tumor may mimic a wide variety of both benign and malignant breast spindle cell lesions, causing a potential diagnostic pitfall. GCT was initially described by Abrikossoff in 1926 [3] and first described in the breast in 1931 [4]. It is composed of tumor cells derived from Schwann cells. GCT consists of compact nests or sheets of cells with an infiltrating growth pattern. The tumor cells demonstrate abundant eosinophilic granular cytoplasm. GCTs must be distinguished from breast cancer, such as the histiocytic variant of invasive lobular carcinoma, apocrine carcinoma, and metastatic neoplasm in the breast that has oncocytic or clear cell features.

Both MFB and GCT typically run an indolent clinical course. Treatment by excision is recommended and curative in both etiologies. No adjuvant therapy is needed. Since both lesions have been reported to mimic carcinoma [5-10], it is imperative for pathologists to pay close attention to the differential diagnosis of these lesions and be able to recognize the benign nature of these entities, further preventing diagnostic mistakes and unnecessary treatment. In order to improve the understanding of both entities and avoid erroneous management, we reported a series of seven patients with these rare breast lesions observed at our institution in the past 10 years.

## Materials and methods

The data for this retrospective study were collected from our database at The University of Texas Medical Branch (UTMB). A series of four cases of GCT and three cases of MFB of the breast were identified between September 2008 and September 2018, each with a histologically confirmed diagnosis. We reviewed the pertinent medical history, imaging findings prior to breast biopsies, histologic features, treatment type, and long-term follow-up.

Breast biopsies were collected from all patients after mammography and ultrasound imaging classified as suspicious or highly suggestive of malignancy by the Breast Imaging Reporting and Data System (BI-RADS ≥ 4A). All hematoxylin and eosin (H&E) and immunohistochemistry stained slides were reviewed to study the morphologic features and their immunoprofiles.

Clinicopathological variables, including patient age and sex, presenting symptoms, imaging findings, histologic findings, and status of the patients at last follow-up, were analyzed. We expressed continuous variables as mean and categorical variables in frequency (percentage).

## Results

Clinical features

Our study consisted of seven subjects: five females and two males. The mean age at onset was 55 years (range from 26 to 86 years). We identified four cases (4/7) with the morphology and immunohistochemical features of GCT and three cases (3/7) were characterized as MFB. Five of the tumors were present on the right and two on the left side of the breast. The mean tumor size was 1.9 cm (ranging from 0.7 to 4 cm). At the time of presentation at UTMB, five patients complained of a palpable nontender mass, one patient complained of breast asymmetry, and one patient was asymptomatic. No symptoms suggestive of breast cancer, including skin retraction, nipple discharge, or axillary node enlargement, were seen in any of our patients (Table 1).

**Table 1:** Clinicopathological and radiological summary of the presented series of patients with myofibroblastoma (MFB) or granular cell tumor (GCT) of the breast.

Patient #	Age/Sex	Histologic diagnosis	Laterality	BI-RADS	Tumor size (mm)	Follow Up (months)	Recurrence
1	52/F	GCT	Right	4B	18	120	No
2	50/F	GCT	Right	4C	9	60	No
3	64/F	GCT	Left	4C	18	21	No
4	34/F	GCT	Right	5	9	1	No
5	86/M	MFB	Right	4A	7	58	No
6	26/F	MFB	Right	4B	40	47	No
7	74/M	MFB	Left	4C	30	5	No

Imaging findings

**Figure 1:**
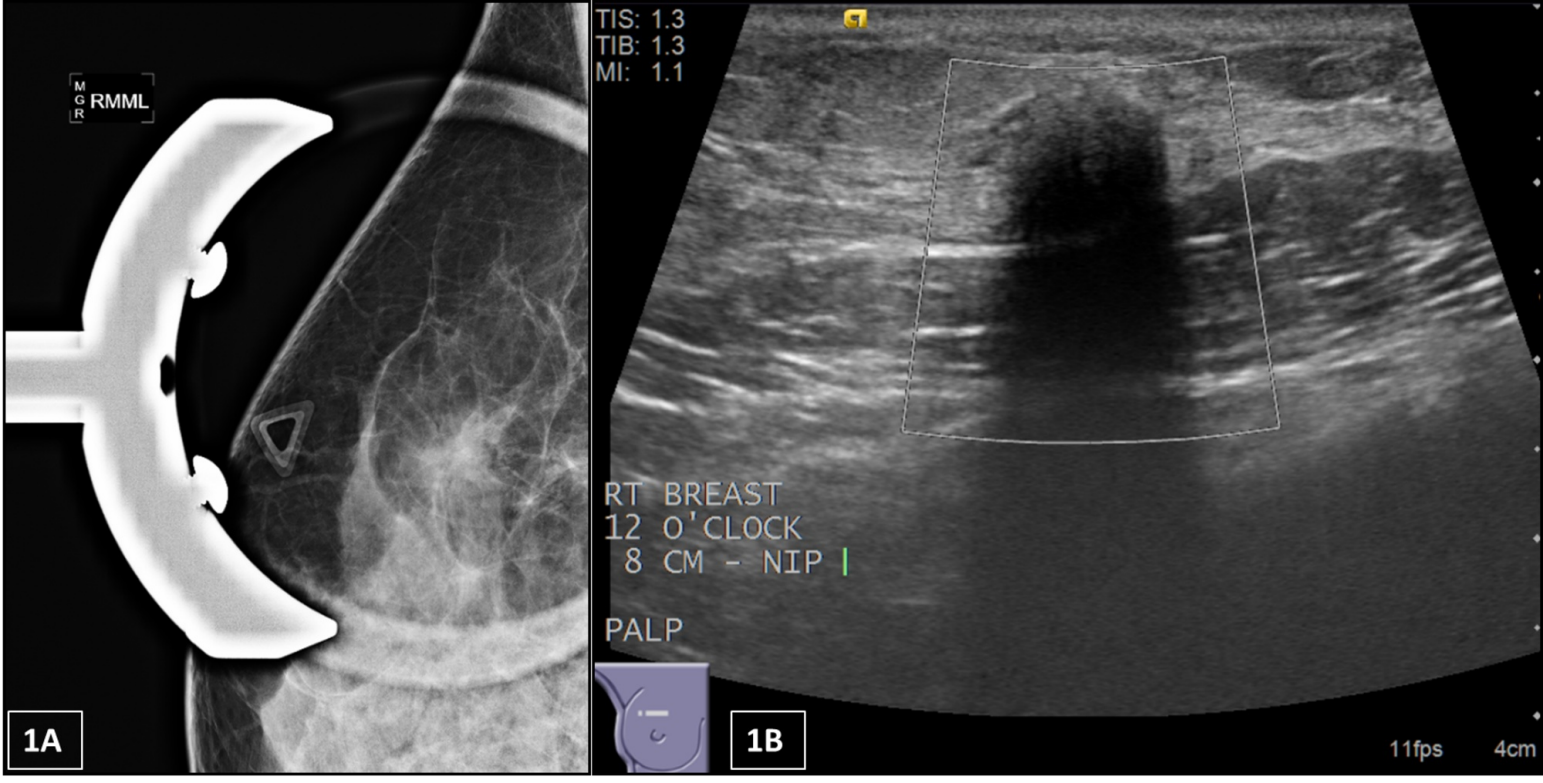
(A) Mammography of the right breast from one of the patients with a granular cell tumor. Triangle marker over the palpable area of concern; there is an irregular mass with indistinct margins. (B) Ultrasound shows an irregular hypoechoic mass with indistinct margins and posterior shadowing with no vascularity measuring 9 x 8 x 8 mm. The radiology categorized this lesion as BI-RADS Category 5 – highly suspicious for malignancy, biopsy recommended. BI-RADS: Breast Imaging Reporting and Data System

All patients had mammography and ultrasound imaging prior to core needle biopsy. From the four patients with histologically proven granular cell tumor (4/7), the ultrasound demonstrated a round or oval solid mass with indistinct or spiculated margins and with a hypoechoic (2/4), anechoic (1/4), or complex (1/4) echo pattern. The radiology categorization before the core needle biopsy for these four patients were: two patients as 4C, one as 4B, and one patient as BI-RADS 5 (Figure 1).

**Figure 2:**
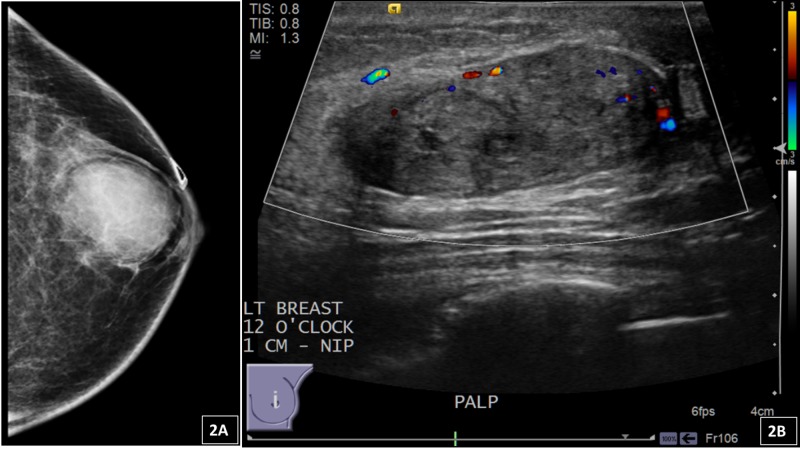
(A) Mammography of the left breast from one of the patients with myofibroblastoma demonstrates an oval mass with circumscribed margins measuring 34 mm. (B) Ultrasound shows an isoechoic oval mass with circumscribed margins and posterior acoustic enhancement. The radiology categorized this lesion as BI-RADS Category 4 B – suspicious for malignancy, biopsy recommended. BI-RADS: Breast Imaging Reporting and Data System

From the three patients with a histologically proven myofibroblastoma (3/7), the mammography/ultrasound showed a round or oval solid mass with circumscribed margins and a hypo (2/3) or hyperechoic (1/3) echo pattern. The radiology categorization pre-core needle biopsy in these cases was: one as 4B, one as 4A, and one patient as BI-RADS 4C (Figure 2).

Gross findings

**Figure 3:**
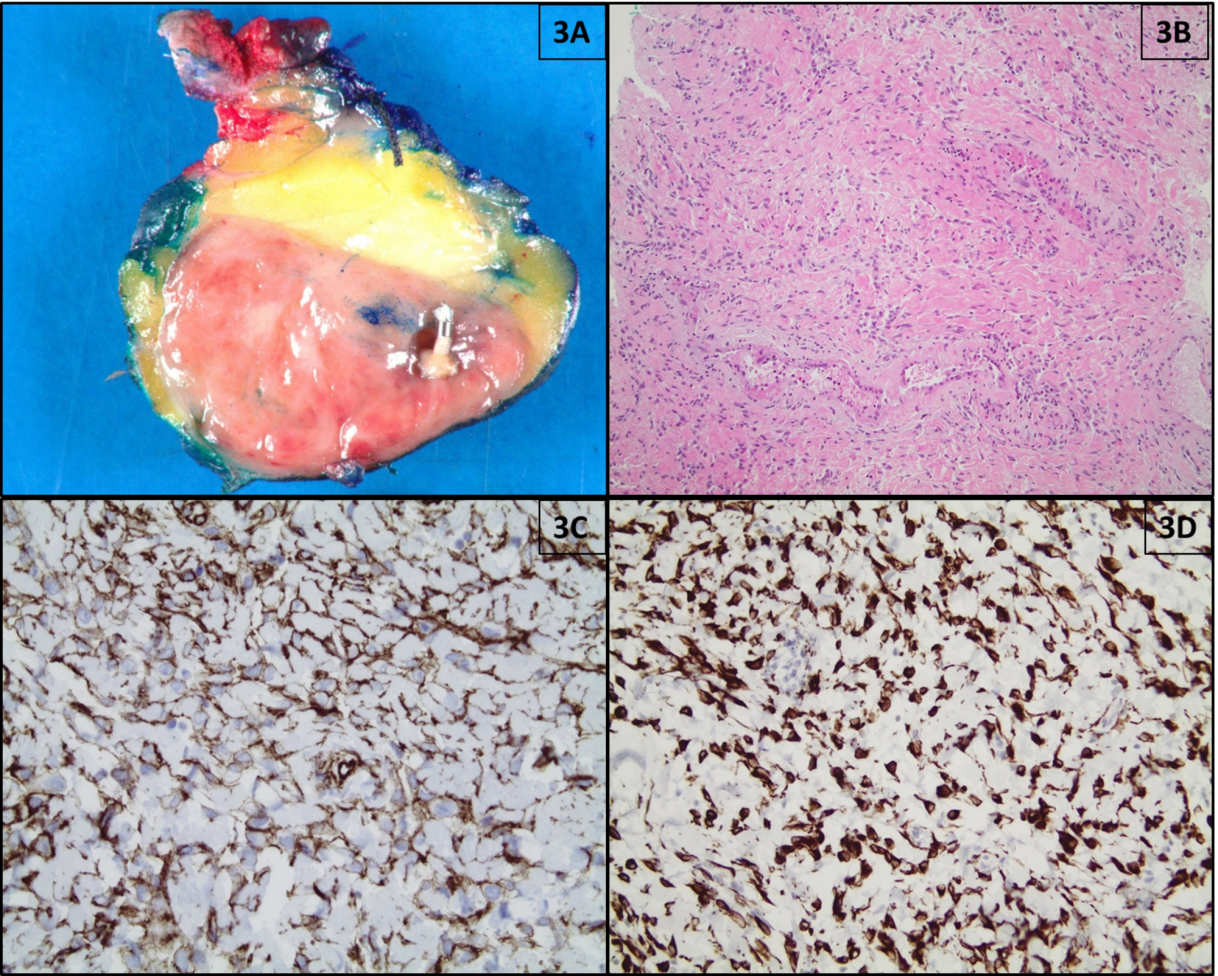
Pathological findings of MFB (patient #7); (A) Resection specimen shows a tan-pink, well-circumscribed, vaguely lobulated solid mass with a metallic clip; (B) Hematoxylin and eosin (H&E) staining shows uniform, slender, bipolar spindle cells haphazardly arranged in small fascicular clusters separated by bands of eosinophilic hyalinized collagen (100x magnification); (C) Tumor cells exhibit immunoreactivity for CD34 by immunohistochemistry (×400 magnification). (D) Tumor cells exhibit immunoreactivity for desmin by immunohistochemistry (×100 magnification). MFB: myofibroblastoma

Three out of four patients with granular cell tumors had an excision following core needle biopsy. And all three myofibroblastoma patients underwent excision. On gross examination, the average tumor size of the surgically excised granular cell tumor was 13.5 mm in the greatest dimension (ranging from 9 mm to 18 mm), containing a tan to white in color, ill-defined, firm mass. The excised myofibroblastomas were tan-pink in color, nodular, and well-demarcated from the surrounding mammary tissue, with a variably whorling appearance. The size of the myofibroblastomas ranged from 7 mm to 40 mm. Necrosis or hemorrhage was not seen in any of the cases (Figure 3A).

Histologic and immunohistochemical findings

Classic myofibroblastomas in two out of three patients consisted of uniform, slender, bipolar spindle cells haphazardly arranged in small fascicular clusters separated by bands of eosinophilic hyalinized collagen. No mitotic figures were found. The tumor in one out of three patients showed a similar morphology with increased cellularity and was called a cellular variant of myofibroblastoma. In all cases, the tumor cells were immunoreactive for CD34, desmin, smooth muscle actin (SMA), and estrogen receptor (ER) and negative for cytokeratin, p63, CD68, and beta-catenin (Figures 3B-3D).

**Figure 4:**
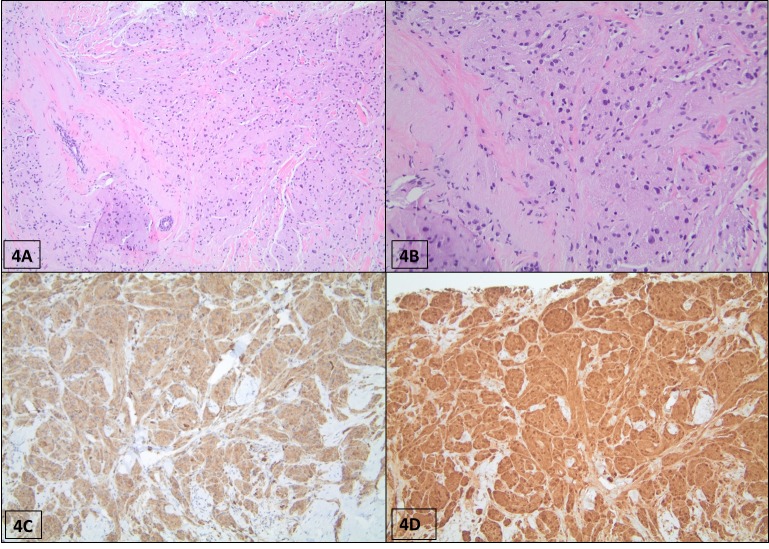
Histopathological section of GCT (patient #4). (A) H&E staining shows nests of tumor cells distributed between collagen bands with ill-defined borders and infiltrative growth into the fat (40x magnification). (B) The tumor cells are large and polygonal with finely granular eosinophilic cytoplasm and round bland nuclei (200x magnification). (C) Tumor cells exhibit immunoreactivity for CD68 by immunohistochemistry (×200 magnification). (D) S-100 staining displaying positive activity on the granular cells, highlighting cytoplasmic granularity (x100 magnification). GCT: granular cell tumor; H&E: hematoxylin and eosin

The granular cell tumors in the four patients were composed of compact nests or sheets of cells that contained abundant eosinophilic and coarse cytoplasmic granules. The cells were polygonal with indistinct cell membranes and well-defined cell borders. The nuclei were round to slightly oval with an open chromatin pattern. The tumors demonstrated an infiltrative growth pattern. No mitotic activity, necrosis, or nuclear pleomorphism was seen. A definitive diagnosis of GCT was established by using immunohistochemistry (IHC). The IHC analysis showed diffuse strong positivity for S100 protein and CD68 (Figures 4A-4D). The cytoplasmic granules were periodic acid-Schiff (PAS) positive and diastase resistant.

Treatment and follow-up

Six patients (6/7, 85.7%) underwent a wide excision following the core needle biopsy diagnosis; five of them underwent lumpectomy and one had a simple mastectomy. One patient was recently diagnosed and has not received surgical excision yet. The prior diagnosis was confirmed for all the cases with a wide excision, and no cases were documented to have positive resection margins. The mean tumor size was 1.9 cm.

The mean follow-up time was 44.5 months (ranging from one to 120 months). None of the lesions recurred or metastasized to the present date.

## Discussion

Benign mesenchymal tumors of the breast are rare and exhibit histological features that can mimic malignant breast lesions. Among them, granular cell tumor and myofibroblastoma are two well-established entities, which are frequently confused on clinical and radiological examination with breast carcinoma [5-10]. Failure to recognize the benign nature of these lesions will result in erroneous patient management and may lead to unnecessary treatment.

Granular cell tumor is a rare condition with a frequency of approximately 1 in 1000 breast tumors. The first report of GCT is attributed to Abrikossoff, who described a GCT as “granular cell myofibroblastoma” [3]. GCT involving the breast accounts for 5% to 6% of GCT cases [4-5]. Because of the positivity of the tumor for S-100 protein, and the similarities of the tumor cells to Schwann cells on electron microscopy, the most widely accepted pathogenetic theory is that the lesion originates from the Schwann cells of interlobular breast tissue [3]. This tumor is more frequent in women between their 40th to 60th decades and is more common in African Americans than in Caucasians [11-12]. All four patients with this type of tumor in our study were female patients with a median age of 55 years; three of them were Caucasians and one was African American.

The major differential diagnostic consideration of GCT on clinical, radiological, and gross examination is invasive carcinoma. Clinically, GCT can mimic malignant disease, sometimes with skin retraction and nipple inversion in superficial lesions. Deep lesions may show fixation to the skeletal muscle of the chest wall and may be adherent to the pectoral fascia. The patients typically present with a palpable, firm, and painless mass, which was noted in three out of four patients with GCT in our study. Radiologically, they typically form a spiculated, irregular, or stellate mass on mammography or solid, poorly defined mass with indistinct margins and posterior shadowing on ultrasound [6,12-16]. From the four patients with histologically proven granular cell tumor, the radiology imaging demonstrated a round or oval solid mass with indistinct or spiculated margins, and with a hypoechoic (2/4), anechoic (1/4), or complex (1/4) echo pattern. In correlation with the cases reported in the literature [10,13-14], our GCT cases were categorized as intermediate to highly suspicious (BI-RADS ≥ 4B) and were mistaken for carcinoma radiologically. One patient was categorized as BI-RADS 5, two patients as 4C, and one as 4B. Due to the great variability in imaging features, the rarity of this tumor in the breast, and not considering it in the differential diagnosis, accurately diagnosing GCT is challenging. On clinical and radiographic examination, it is virtually impossible to establish a definitive diagnosis of GCT. Therefore, ultrasound-guided core biopsy is necessary to make the definitive diagnosis.

Macroscopically, GCT demonstrates as a gray-white to tan, firm mass, and some tumors show ill-defined borders and may infiltrate, as in our cases, into the surrounding tissues. These features may mimic malignant growth patterns. Microscopically, GCTs must be distinguished from breast cancer, especially from a histiocytic variant of invasive lobular carcinoma, apocrine carcinoma, histiocytic lesions, as well as a metastatic neoplasm in the breast that has oncocytic or clear cell features, such as renal carcinoma, malignant melanoma, and alveolar soft part sarcoma [5,11,17]. GCTs are composed of compact nests or sheets of cells with an infiltrating growth pattern. The cells are polygonal or spindle-shaped with well-defined borders, abundant eosinophilic granular cytoplasm, and small uniform nuclei with open chromatin. The granular change is caused by the cytoplasmic accumulation of lysosomes. No mitoses or cytological atypia are seen in GCT, which helps to differentiate it from malignant neoplasm [4-5,11,13]. GCT has a spectrum that ranges from benign to atypical to malignant. Malignant GCT may occur (less than 1%) and specific diagnostic criteria must be met to be considered as such [18-19]. Six histological criteria were proposed for a distinction between benign and malignant GCTs. These criteria are necrosis, spindling, vesicular nuclei with large nucleoli, increased mitotic activity (>2 mitoses per 10 high power ﬁeld at 200 magniﬁcation), high nuclear/cytoplasmic ratio, and nuclear pleomorphism. These criteria classify GCT histologically into atypical (when two of these six criteria are present) and malignant (when three or more criteria are present) [11,20].

Immunohistochemistry helps to distinguish GCT from invasive mammary carcinomas, including apocrine carcinoma. The tumor cells show positive immunoreactivity against S100 protein, CD68, carcinoembryonic antigen (CEA), and vimentin. Cytokeratin staining is negative and most lesions are negative for estrogen, progesterone receptors, and androgen receptor. The granular cytoplasm is positive for PAS and is diastase resistant. In our presented cases, the pathological features were supported by IHC, as reported in the literature [4-6,11-12].

MFB is a rare benign spindle cell tumor of mammary stroma composed of myofibroblasts. This entity was first described by Wargotz et al. [1]. The prevalence of MFB in the breast was shown to be approximately 10% [2]. The original report demonstrated that MFB had a male predominance. However, subsequent studies illustrated that it can occur nearly equally in both sexes and over a wide age range [1-2,21-23]. Our study consisted of two males and one female and a wide age range, which is consistent with the literature. Clinically, MFB typically presents as a circumscribed, mobile, painless, solitary mass that usually grows slowly. This presentation was found in our patients. Radiologically, mammography or ultrasonography typically shows a well-circumscribed and homogeneous, slightly hypoechoic, solid mass devoid of microcalcifications. Our three patients with a histologically proven myofibroblastoma (3/7) showed a round or oval solid mass with circumscribed margins by mammography/ultrasound. One patient was categorized as BI-RADS 4C, one as 4B, and one as 4A.

Macroscopically, MFB tumor size can range from a few millimeters to 11 cm and is generally a well-circumscribed, ﬁrm, and rubbery, unencapsulated, round to oval mass. In some cases, the tumor may show focal to extensive mucoid- or lipomatous-appearing areas. However, cystic degeneration, necrosis, and hemorrhage are not features of MFB. The size of our MFB cases measured from 7 mm to 40 mm. The excised tumors were tan-pink in color, nodular, and well-demarcated from the surrounding mammary tissue, with a variably whorling appearance. Neither necrosis nor hemorrhage was seen in any of our cases.

MFB may exhibit a wide spectrum of histological features and varied cellularity that can be misinterpreted as a malignant tumor. Histologically, the classic type of MFB is a circumscribed tumor consisting of uniform spindle cells with bland, oval nuclei and indistinct borders that are arranged in short fascicles and admixed with bands of eosinophilic hyalinized collagen [1,21,24-25]. In addition to the classical type of myofibroblastoma, which in our study was identified in both male patients (Table 1, patients #5 and #7), several unusual morphologic variants, such as the cellular, infiltrative, epithelioid, deciduoid-like, lipomatous, collagenized/fibrous, and myxoid variants have been described [8]. The lack of marked cytological atypia, along with the absence of necrosis and mitotic activity in the classic type of MFB, help to verify its benign nature. However, the diverse and complicated variants of MFB lead to diagnostic challenges for pathologists. One of the patients with MFB in our study is a young woman who presented with the cellular variant (Table 1, patient #6). The differential diagnoses of MFB are broad and include reactive processes and benign neoplasms, such as a solitary fibrous tumor, nodular and proliferative fasciitis, fibromatosis, spindle-cell lipoma, myoepithelioma, and pseudoangiomatous stromal hyperplasia [8,21]. Malignant neoplasms, such as stromal sarcoma, malignant fibrous histiocytoma, and spindle-cell or metaplastic carcinoma, should not be confused with a myofibroblastoma [8,26-27].

Immunohistochemistry may be necessary to arrive at a correct diagnosis. The MFB tumor cells show immunoreactivity for CD34, desmin, and, variably, with smooth muscle actin (SMA), estrogen receptor (ER)/progesterone/androgen receptor, CD99, B-cell lymphoma 2 (Bcl2), and CD10 [8,21,28], and they do not express cytokeratins or p63. MFB has genetic rearrangement or deletion of 13q14, resulting in loss of Rb expression by IHC [2]. The IHC pattern can help to distinguish MFB from other entities. In all of our MFB cases, the tumor cells were immunoreactive for CD34, desmin, SMA, and ER and negative for cytokeratin, p63, CD68, and beta-catenin.

The clinical significance of these entities lies primarily in its recognition as a distinctive benign neoplasm. It is important to make a deﬁnitive diagnosis preoperatively to avoid extensive resection and axillary clearance for carcinoma. For both lesions, complete local excision with free margins is the treatment of choice [16]. Sentinel lymph node biopsy is not indicated. No specific adjuvant therapies are suggested; however, long-term follow-up is strongly recommended [27]. Local recurrence may occur after incomplete excision. However, the risk of recurrence is extremely low and has been reported to be less than 1.5% in MFB cases [2].

## Conclusions

Our cases illustrate that although GCT and MFB of the breast are relatively rare breast neoplasms, they should always be considered in the differential diagnosis of benign and malignant lesions. Since clinically and radiologically these lesions can mimic invasive carcinoma and the definitive diagnosis is made by pathology, pathologists should bear this in mind to avoid misdiagnosing breast carcinoma, which could lead to unnecessary surgery.
